# Hypoxia-inducible ERO1α promotes cancer progression through modulation of integrin-β1 modification and signalling in HCT116 colorectal cancer cells

**DOI:** 10.1038/s41598-017-09976-7

**Published:** 2017-08-24

**Authors:** Norio Takei, Akihiro Yoneda, Kaori Sakai-Sawada, Marina Kosaka, Kenjiro Minomi, Yasuaki Tamura

**Affiliations:** 1 Department of Molecular Therapeutics, Center for Food and Medical Innovation, Institute for the Promotion of Business-Regional Collaboration, Hokkaido University, Kita-21 Nishi-11, Kita-ku, Sapporo, 001-0021 Japan; 2 Research & Development Department, Nucleic Acid Medicine Business Division, Nitto Denko Corporation, Osaka, Japan

## Abstract

Endoplasmic reticulum disulphide oxidase 1α (ERO1α) is an oxidase localized in the endoplasmic reticulum that plays a role in the formation of disulphide bonds of secreted and cell-surface proteins. We previously showed that ERO1α is overexpressed in various types of cancer and we further identified ERO1α expression as a novel factor related to poor prognosis in cancer. However, the biological functions of ERO1α in cancer remain unclear. Here, we investigated the cell biological roles of ERO1α in the human colon-cancer cell line HCT116. ERO1α knockout (KO) by using CRISPR/Cas9 resulted in decreased tumourigenicity *in vivo* and reduced cell proliferation only under hypoxia *in vitro*, which suggested that ERO1α promotes cancer progression specifically in a low-oxygen environment. Thus, we evaluated the function of ERO1α in cell proliferation under hypoxia, and found that under hypoxic conditions, ERO1α KO resulted in a contact-inhibited morphology and diminished motility of cells. We further showed that ERO1α KO induced a change in integrin-β1 glycosylation and thus an attenuation of cell-surface integrin-β1 expression, which resulted in the aforementioned phenotype. Our study has established a previously unrecognized link between ERO1α expression and integrin activation, and thus provides new evidence for the effectiveness of ERO1α-targeted therapy for colorectal carcinoma.

## Introduction

The endoplasmic reticulum (ER) is an organelle in which newly synthesized secreted proteins and membrane proteins acquire their proper conformation and posttranslational modification, and thus a stringent protein quality-control system operates in the ER^1^. For example, the ER harbours several oxidizing enzymes that act cooperatively in the formation of protein disulphide bonds, although the main functional pathway in this case is the oxidation pathway of ERO1-PDI (ER disulphide oxidase-protein disulphide isomerase)^2^, which includes the oxidizing enzyme ERO1α. Intriguingly, despite functioning in the ER, ERO1α lacks the ER-localization signals (such as a C-terminal KDEL sequence) that are present in other oxidizing enzymes. Therefore, ERO1α behaves in a manner distinct from other redox enzymes^3^.

Previously, we reported that ERO1α expression is higher in various types of cancer cells than in normal cells, and that the expression level is associated with cancer progression and prognosis^4, 5^. We further showed that ERO1α facilitated disulphide bond formation in granulocyte-colony stimulating factor^6^. Moreover, other reports have also indicated that ERO1α is involved in regulating the expression of tumour growth factors such as VEGF^4, 7^. Thus, ERO1α overexpression is considered to play a key role in cancer biology, and given that its expression level is positively correlated with cancer exacerbation, ERO1α could serve as a suitable target in cancer therapy. However, the mechanistic underpinnings of ERO1α function in cancer biology remain unclear and warrant detailed analysis.

Here, we investigated the effect of ERO1α gene deletion on cancer biology *in vitro* and *in vivo*. We found that ERO1α plays a critical role in integrin glycosylation and membrane transport in hypoxic tumour cells, and that ERO1α deletion leads to attenuated integrin signalling, including in tumour growth and epithelial-mesenchymal transition (EMT).

## Results

### Establishment of ERO1α knockout (KO) HCT116 cells

**Figure 1:**
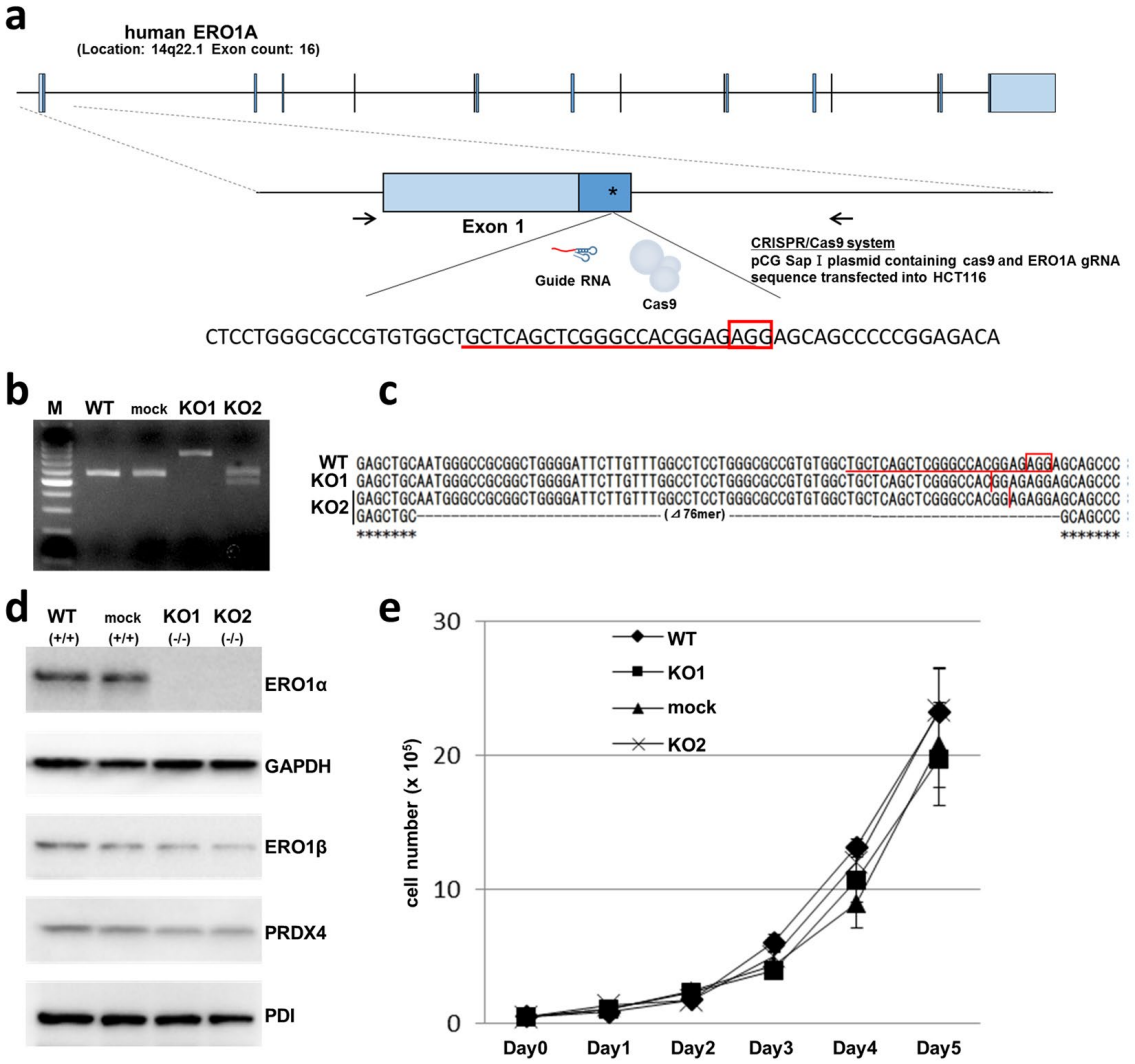
*ERO1A* gene deletion in HCT116 cells. (**a**) The human *ERO1A* gene is located on chromosome 14. Exons are represented as boxes. The asterisk indicates the region targeted by the gRNA, and the arrows indicate the PCR primer set. (**b**) Two representative mutated alleles in the HCT116 cell line induced by CRISPR/Cas9, as detected using RT-PCR. (**c**) CRISPR/Cas9-induced deletion is shown using dashed lines; the red vertical bar indicates the site of the insertion sequence. In KO-1, the origin of the pBluescript backbone sequence (236 bp) was inserted in the gRNA target locus. KO-2 contained a 76-bp deletion and a 1-bp (G) insertion (flame-shift mutation). (**d**) Western blotting analysis of the expression of ERO1α and related proteins: comparison of ERO1α KO clones with WT and mock control. (**e**) Cell growth in each clone was assessed by counting cells at the indicated time points; data represent the means ± s.e.m. (*n* = 6). The groups showed no statistically significant differences.

ERO1α−/− (KO) clones were developed in HCT116, a human colorectal carcinoma cell line, by using the CRISPR/Cas9 system (Fig. 1a). Based on mutation detection in the T7 endonuclease assay, we confirmed the cleavage of several clones, which indicated that the targeted alleles were deleted in these clones. Sequence analyses revealed that most clones were biallelic or harboured heterogeneic mutations. The mutations ranged from the insertion of a few hundred base pairs derived from the introduced pBluescript origin, to the deletion of a few or several tens of base pairs near the gRNA target locus. From these clones, we selected two appropriate clones carrying the insertion of the pBluescript backbone sequence or lacking a few tens of base pairs (Fig. 1b and c), and used these clones in our experiments.

The lack of ERO1α protein expression in the obtained KO clones was confirmed through western blotting (Fig. 1d). As controls, we used wild-type (WT) and a mock control in which the empty vector alone was introduced (as a negative control). In the KO clones, other oxidoreductases were expressed at a level comparable to that in WT, which indicated that the targeting of the ERO1α allele did not affect the expression of these other enzymes (Fig. 1d). Moreover, the *in vitro* cell proliferation of the ERO1α KO clones did not differ from that of either WT or the mock clone (Fig. 1e).

### *In vivo* analysis of ERO1α KO cell tumourigenicity

**Figure 2:**
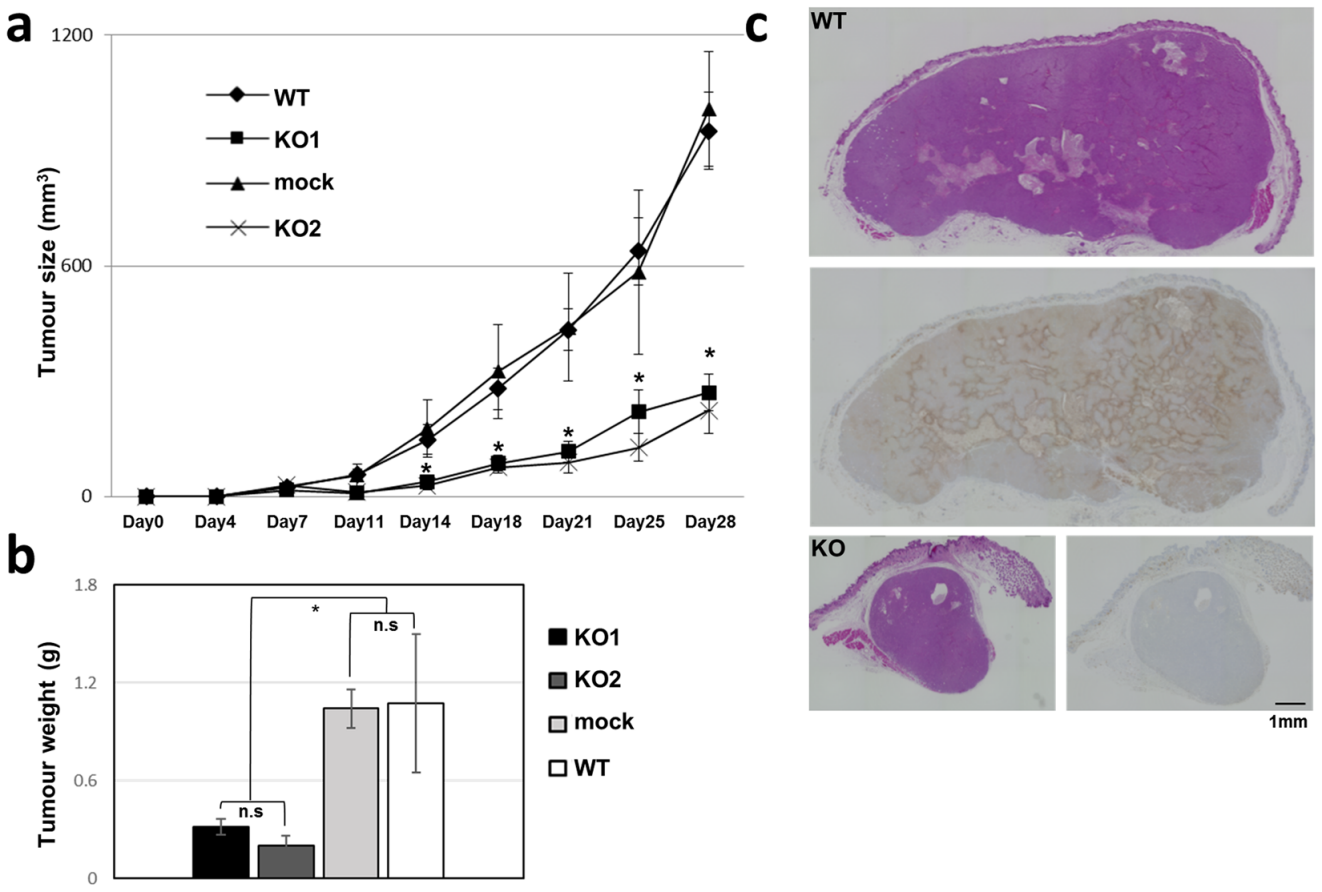
Effect of ERO1α KO on xenograft tumour growth. (**a**) Tumour size was measured twice a week after subcutaneous injection of control and KO cells. (**b**) Tumour weight was measured after dissecting out the tumours; values shown are the means ± s.e.m. (*n* = 8). *P < 0.05, n.s, not significant (Student’s *t* test). (**c**) Representative image of haematoxylin and eosin staining and immunohistochemical staining against ERO1α.

To confirm the tumourigenicity of the two selected KO clones, we conducted xenograft experiments in BALB/c nu/nu mice. In the KO-cell groups, the tumour was engrafted (Fig. 2a and b), but the tumourigenicity here was markedly diminished as compared with that of the WT or mock control group, which suggested that ERO1α deficiency reduced the tumourigenicity of the cancer cells. We also immunostained the excised tumours, and the results showed that ERO1α-positive cells in the WT group were confined to the margin of the tumour and the area near sites of necrosis (Fig. 2c); this staining is generally within a region that is considered to be low in oxygen. These results indicate that ERO1α-positive cells present a characteristic cancer-cell proliferation phenotype and are localized in a region where invasion and metastasis are activated.

### ERO1α KO cells exhibit reduced growth under hypoxia

**Figure 3:**
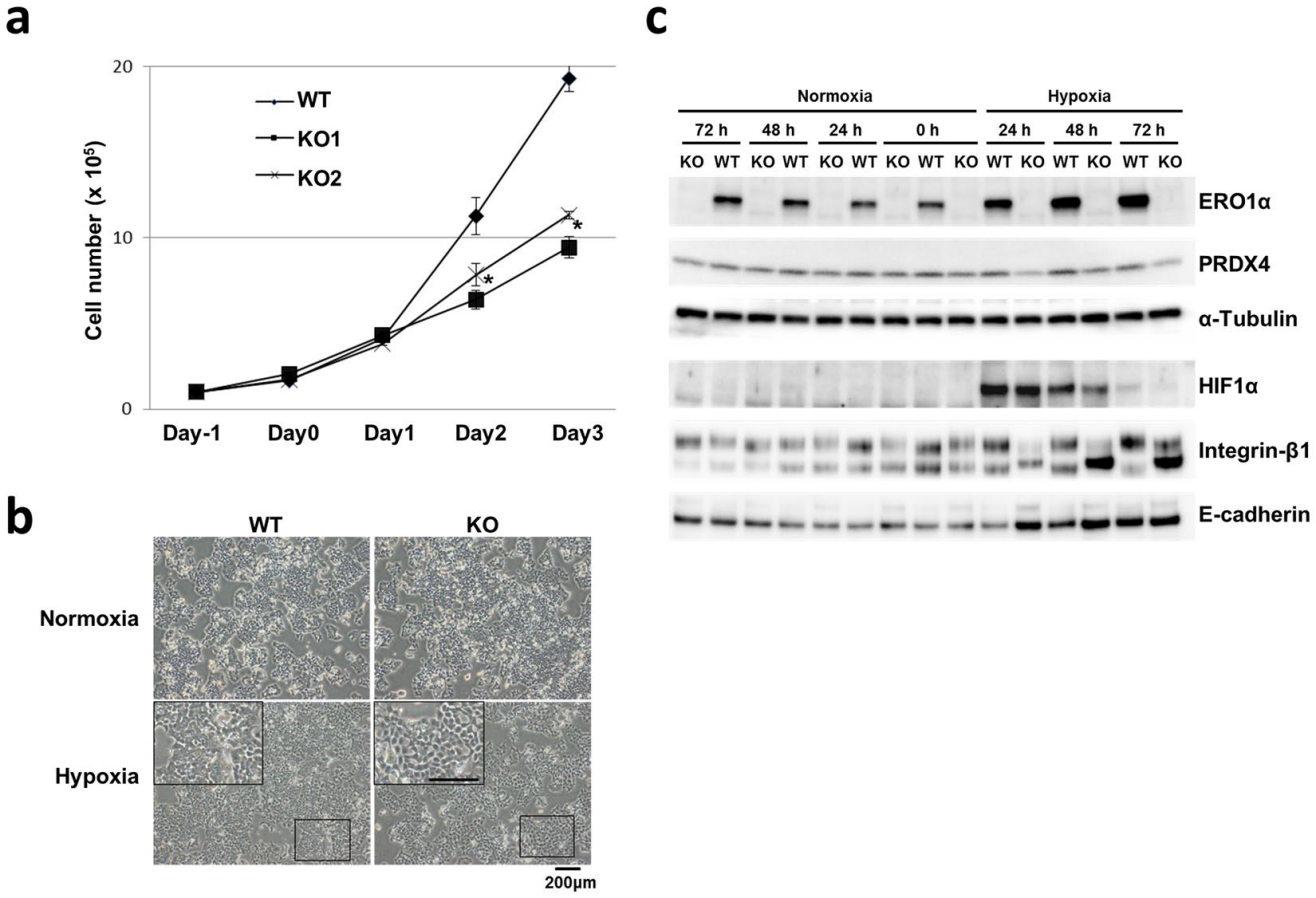
*In vitro* culture of WT and ERO1α cells under normoxia vs hypoxia. Cultures were incubated for 24, 48, and 72 h under hypoxia or normoxia. (**a**) Cell-proliferation curve of WT and KO clones under hypoxia, assessed by counting cells at the indicated times. Data are shown as the means ± s.e.m. (*n* = 4). *P < 0.05, Student’s *t* test. (**b**) Representative light microscopy images of WT (left) and KO (right). The KO clone displays reduced stacking (piling up) and increased cell-cell integrity as compared with WT. (**c**) Western blotting analysis of oxidizing enzymes and cell adhesion molecules. Under hypoxia, ERO1α depletion impairs the maturation of integrin-β1. The lower integrin band (110 kDa) represents the β1 precursor, and the upper (approximately130 kDa) the mature form. All experiments were conducted at least three times and representative data is presented.

Next, we analysed the mechanism by which tumourigenicity is weakened owing to ERO1α deficiency in xenografts. We focused on the effect of a hypoxic-stress environment based on considering the microenvironment involved in tumour formation, and we compared the WT with the KO clones in hypoxic cultures prepared in a low-oxygen incubator. The KO clones did not exhibit reduced cell proliferation as compared with WT in normoxic cultures, but their proliferation was markedly decreased under hypoxia (Fig. 3a). Moreover, bright-field microscopy analysis revealed clear morphological differences between WT and KO clones under hypoxic but not normoxic conditions; the KO clones displayed higher cell-cell integrity (contact inhibition) and reduced ‘piling up’ relative to WT (Fig. 3b).

### Integrin maturation is attenuated in ERO1α KO cells under hypoxia

The aforementioned results showed that under hypoxia but not normoxia, the KO cells displayed morphological changes, such as diminished piling up, whereas the WT cells were stacked on the culture dishes and atop other cells even under hypoxia. Thus, in the hypoxic cultures, individual WT cells could not be readily distinguished from each other; by contrast, the morphology of individual KO cells was discernible despite the cells growing in close contact with each other, and the stacking here was sparse. To elucidate the molecular changes involved in this suppression of cell proliferation of the KO clones under hypoxia, we analysed cell adhesion molecules (CAMs) such as E-cadherin and integrin-β1 (which interacts with several integrin α subunits). Our results indicated that integrin-β1 protein levels were not altered under normoxia, but the amount of the mature form of the protein was decreased under hypoxia in the KO clones and that of the immature form was increased (Fig. 3c). In comparison, the gene expression level of integrin-β1 was analysed by qPCR and microarray, which demonstrated no significant difference between WT and KO (Supplementary Fig. S1a and b).

### ERO1α deficiency impairs integrin-β1 N-glycosylation and membrane trafficking under hypoxia

**Figure 4:**
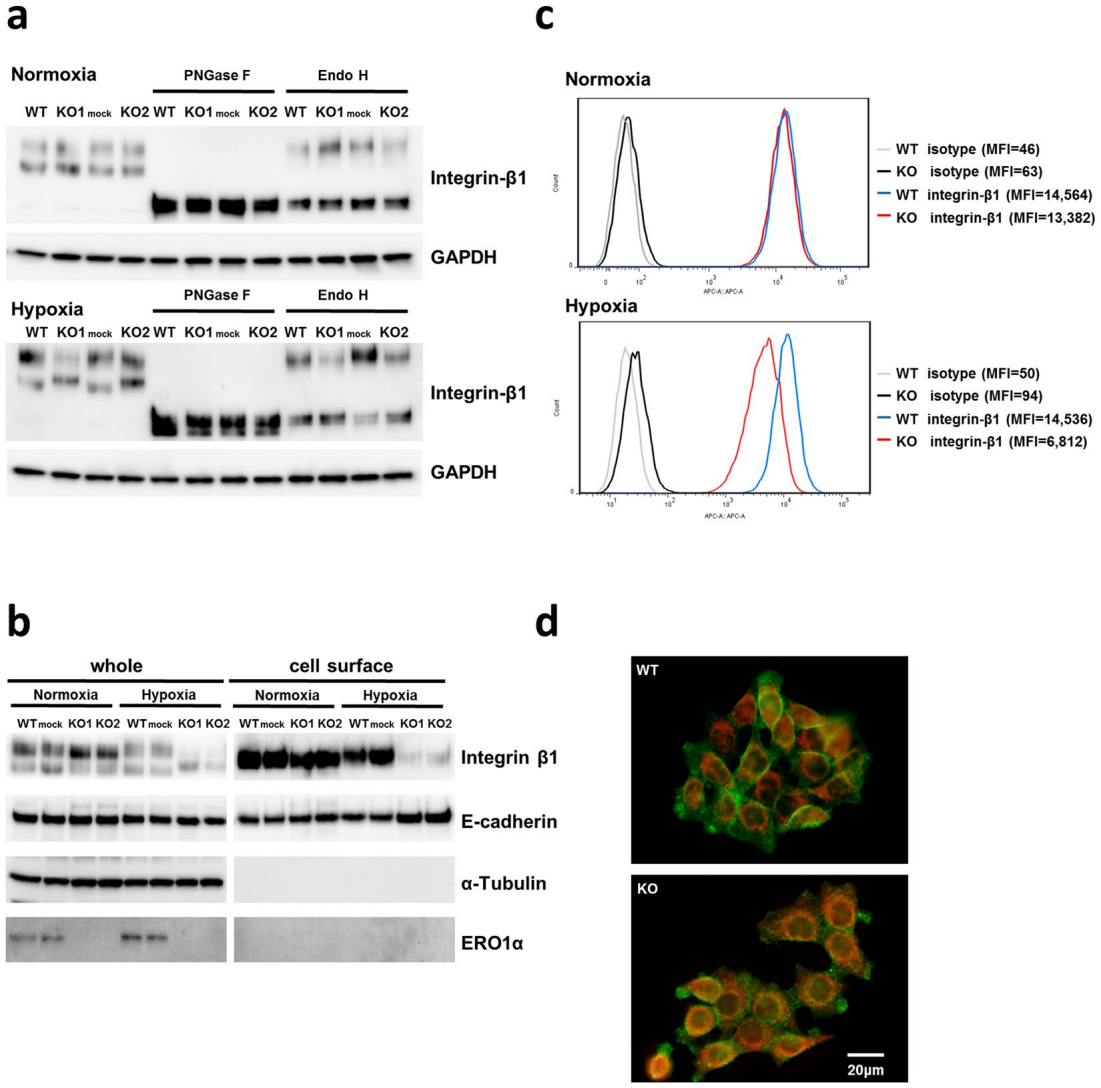
ERO1α deletion drastically reduces the cell-surface-expressed form of integrin-β1 under hypoxia. (**a**) Normoxic and hypoxic samples were incubated without or with Endo H or PNGase F. ERO1α depletion impaired Endo H-resistant N-glycosylation of integrin-β1 under hypoxia. Membrane integrin-β1 levels in HCT116 clones under normoxia and hypoxia were assessed by performing cell-surface biotinylation assays, flow cytometry, and immunofluorescence labelling of cells: (**b**) Cell-surface biotinylation assay performed on WT and the mock and KO clones under normoxia and hypoxia. Total lysates (whole) and the NeutrAvidin agarose-bound fraction (cell surface) were subjected to western blotting. (**c**) Flow cytometric analysis of integrin-β1 in WT and KO. Cells were collected and incubated with APC-conjugated anti-integrin-β1 antibody or isotype control antibody. FMI, Fluorescence mean intensity. (**d**) Immunocytochemical labelling of integrin-β1 (green) and PDI (red) in WT and KO cells under hypoxia. PDI was used as an ER marker protein. All experiments were conducted in duplicate or triplicate and repeated at least twice.

To ascertain whether the integrin-β1 change observed in ERO1α KO cells under hypoxia was caused by impaired N-glycosylation, we performed western blotting on lysates treated with endoglycosidase H (Endo H) or peptide N-glycosidase F (PNGase F) (Fig. 4a). Whereas the lower integrin-β1 species was completely digested by Endo H to the size of the core protein, the upper β1 band, which is glycosylated in the process of transport from the ER to medial-Golgi apparatus, was Endo H-resistant^8^. Notably, the levels of the upper band were decreased in the KO-clone cells cultured under hypoxia, but the core protein of integrin-β1, which was detected after PNGase F digestion, showed no difference (Fig. 4a).

Next, we examined whether the level of the upper integrin-β1 species was decreased because of an inhibition of transport to the cell surface, which we evaluated by performing cell-surface biotinylation and western blotting, flow cytometry, and immunocytochemical analyses (Fig. 4b–d): The 130-kDa mature integrin-β1, but not the 110-kDa immature protein, was detected among cell-surface biotinylated proteins, and the cell-surface expression of integrin-β1 was found to be lower in KO cells than in WT cells under hypoxia but not normoxia; moreover, integrin-β1 in the hypoxic KO cells was localized in the ER. Thus, the transport of the fully mature form of integrin-β1 to the cell membrane was impaired in the KO clones under hypoxia.

### ERO1α KO induces integrin-β1 inactivation and affects the expression of EMT-related proteins under hypoxia

**Figure 5:**
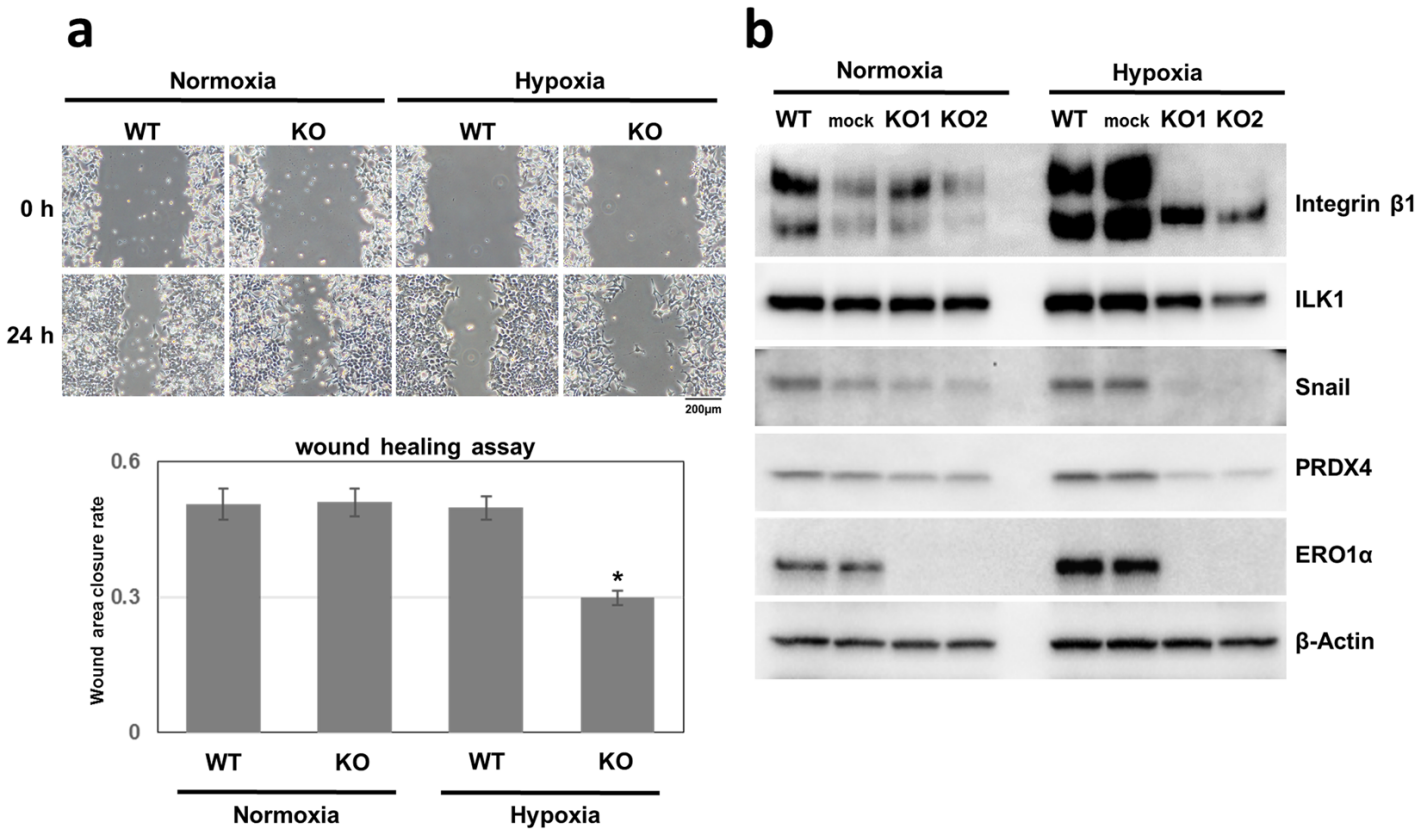
ERO1α deletion affects cell motility and integrin- and EMT-related signalling molecules under hypoxia through integrin-β1 downregulation. (**a**) Wound-healing assays performed using the indicated cell clones and culture conditions. Results are given as the means ± s.e.m. (*n* = 4); *P < 0.05, Student’s *t* test. The upper panels show the wound at baseline, the lower the wound after 24 h. Under hypoxia, the KO clone exhibited significantly decreased migration as compared with WT and with KO under normoxia. (**b**) Expression of integrin- and EMT-related molecules in WT and the mock and KO clones under normoxia and hypoxia.

The morphological changes and attenuated cell-surface integrin-β1 expression in the KO clones under hypoxia suggested a potential alteration in cell migration, which involves integrin signalling. Thus, we next assessed cell migration by performing wound-healing assays. The wound-closure rates measured at 24 h indicated that the KO-clone cells showed diminished migration capacity relative to control cells under hypoxia but not normoxia (Fig. 5a).

Integrin-β1 KO in a breast carcinoma cell line was previously reported to inhibit the expression of EMT-related molecules^9^. Therefore, we predicted that ERO1α KO would influence the expression of EMT-related molecules through the effect on integrin modification under hypoxia, and we confirmed this by examining the expression of EMT-related proteins: ERO1α KO clones exhibited an increase in E-cadherin levels (Fig. 3c) but a reduction in the expression of Snail and also in that of integrin-linked kinase 1 (ILK1) (Fig. 5b), which is involved in the integrin signalling leading to the EMT cascade.

### *ERO1A* gene introduction reverses the effects of ERO1α KO

**Figure 6:**
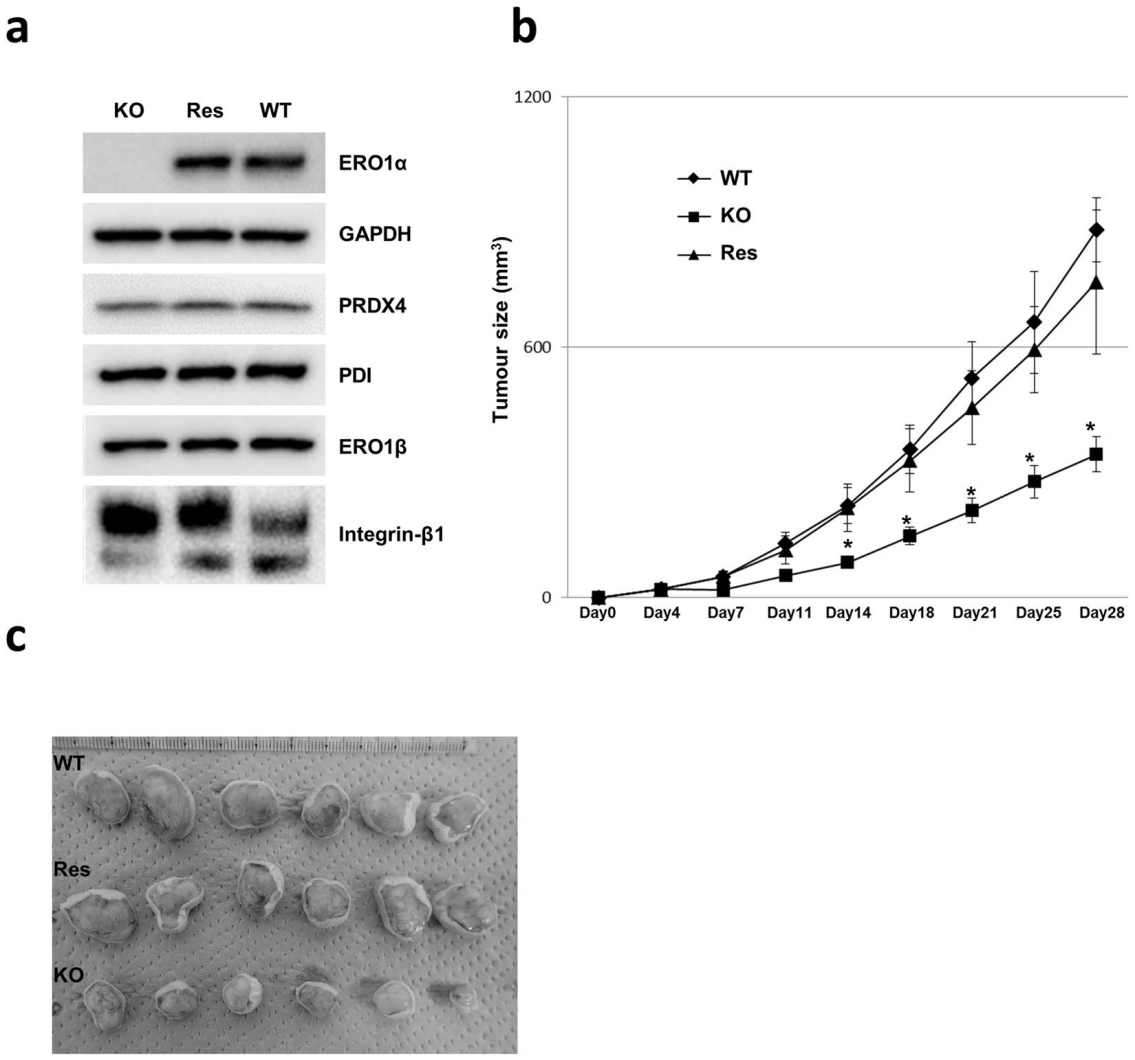
ERO1α re-expression reverses the effect of ERO1α KO. (**a**) Western blotting to confirm ERO1α expression. Res: ERO1α-transfected KO clone. (**b**) Tumour size was measured twice a week after subcutaneous injection; *n* = 6, *P < 0.05 (Student’s *t* test). (**c**) Images of tumours at 28 days after inoculation with the indicated clones.

To directly verify that the phenomena described in the preceding subsections were due to ERO1A gene depletion, we established an ERO1α-rescued cell clone. A plasmid carrying the ERO1α gene (Origene) under the control of CMV promoter was introduced into a KO clone, and after selection for puromycin-resistance, the ERO1α-rescued clone was established. The results of western blotting performed using lysates from cells cultured under normoxia showed that ERO1α was expressed in the rescued clone at almost the same level as ERO1α in WT cells (Fig. 6a). Next, we confirmed *in vivo* phenotype recovery by using this clone in our xenograft model. The results showed that the rescued clone exhibited drastically higher tumourigenicity as compared with the KO clone, and that the tumourigenicity of the rescued clone was comparable to that of WT, which indicated that exogenous ERO1α expression caused the recovery of WT levels of tumourigenicity in the KO clone (Fig. 6b and c).

## Discussion

In this study, we focused on ERO1α, a protein that is overexpressed in cancer cells. To uncover the importance of ERO1α expression in cancer biology and the molecular mechanisms underlying its protein functions, we generated conventional ERO1α KO clones and used the clones to perform mechanistic analyses. We established the ERO1α KO colorectal cancer cell lines by using the CRISPR/Cas9 system; among the genetic-modification techniques that trigger non-homologous end joining, the CRISPR/Cas9 system is more specific than the methods employing zinc finger nucleases or transcription activator-like effector nucleases. In the obtained clones, in which ERO1α exon 1 was specifically targeted, ERO1α protein expression was eliminated. Moreover, the accurate gene editing possible with the CRISPR/Cas9 system enabled these ERO1α−/− clones to be established without any effect on ERO1β, an ERO1α paralog.

Examination of the morphology and the *in vitro* proliferation ability of the ERO1α−/− clones in the HCT116 background revealed that the clones appeared healthy and that in these phenotypic aspects, the clones were no different from the WT and mock control clones. However, in a xenograft model, the tumourigenicity of the KO clones was unexpectedly found to be markedly diminished relative to the control. This finding led us to conclude that ERO1α predominantly functions in specific circumstances, such as hypoxia, which is one of the key factors related to the cancer microenvironment. Thus, we performed *in vitro* proliferation assays on normoxic and hypoxic cultures, with our results showing that the proliferative capacity of the WT and KO cells differed markedly only in the hypoxic environment and that the ERO1α KO cells exhibited attenuated proliferation. In addition, under hypoxia, the ERO1α−/− cells also exhibited a morphological change, maintaining their cell-cell integrity. Therefore, we next focused on the loss of contact inhibition, a characteristic phenotype of cancer cells, and sought to detect changes in the CAMs involved in contact inhibition.

Contact inhibition is widely recognized to be regulated through a coordinated crosstalk between integrins and cadherins^10^ with phenotypes that compromise cadherin adhesive activity or localization at cell membranes correlating with diseases such as cancer^11–14^. Moreover, E-cadherin upregulation has been reported to induce contact inhibition^15^. Conversely, integrins are involved in vascular proliferation, adhesion, and wound repair, and changes in the expression of integrins have been reported to lead to morphological alterations, particularly in the case of integrin-β1 expression^16^.

As noted above, based on considering the morphological change observed in the KO cells under hypoxia, we examined ECM-related molecules, particularly integrins among the CAMs. Furthermore, given that tumourigenesis was attenuated *in vivo* in the case of the KO clones, we specifically analysed the expression level of integrin-β1, which interacts with a wide range of partners among the integrin-family proteins. Our results showed that integrin-β1 glycosylation was altered under hypoxia in the ERO1α KO clones. Protein glycosylation is a major posttranslational modification that is associated with diverse biological processes, because glycosylation controls protein folding, localization, stability, and interaction. In the case of integrin-β1, N-glycosylation of the protein has been reported to be critical in cell adhesiveness and motility^17^.

In the course of protein synthesis, even after proteins are secreted into the Golgi apparatus, the proteins that should be localized in the ER or the immature proteins that fail to adopt their final proper conformation are transported back to the ER by COPI-coated vesicles^3^. Previous studies have revealed that inhibition of disulphide bond formation through PDI depletion impairs protein export from the ER to the Golgi^18^, and that Endo H-resistant glycan structures can be examined to monitor protein translocation through the Golgi^19^. Thus, we suspected that aberrant integrin-β1 glycosylation in the KO cells could attenuate transport of the protein to the cell membrane; to test this, we evaluated the integrin-β1 glycosylation pattern and the level of cell-surface transport. Our results showed that the amount of Endo H-resistant glycosylation and the cell-surface expression of integrin-β1 were decreased in the KO cells under hypoxia, which suggested that under this condition, the lack of ERO1α led to an inhibition of cell-surface transport of integrin owing to failure of protein folding during maturation. Moreover, as expected here, immunocytochemical analyses confirmed integrin-β1 accumulation in the ER. This result is similar to the results in a previous report^20^. In contrast, under normoxia, integrin-β1 was transported to the cell membrane in the KO cells as in control cells, likely because other oxidizing enzymes such as ERO1β and peroxiredoxin 4 (PRDX4) compensated for the lack of ERO1α. Thus, under hypoxia, ERO1α predominantly regulates integrin-β1, with absence of the enzyme resulting in drastic attenuation of integrin-β1 glycosylation and cell-surface transport.

In order to confirm whether similar phenomena could be obtained in other colon cancer cell lines, a KO clone was established using the HT-29 human colorectal adenocarcinoma cell line (Supplementary Fig. S2a) and the tumourigenicity and integrin-β1 behaviour were confirmed. Similar to results obtained with the HCT116 clone, attenuated tumourigenicity (Supplementary Fig. S2b–d) and reduced cell surface localization of integrin-β1 was confirmed in HT-29 KO clones (Supplementary Fig. S2e). Thus, overall, our findings indicate that ERO1α plays a critical role in hypoxic cancer biology, because among oxidizing enzymes, ERO1α is predominantly upregulated under hypoxia^21, 22^. Notably, in this regard, another oxidoreductase, PRDX4, was found to be downregulated under hypoxia in this study (Figs 3c and 5b).

Integrin signalling through ILK has been reported to upregulate the transcriptional repressors SNAIL/SLUG and subsequently suppress E-cadherin transcription^23–25^. Accordingly, our results indicated that the decrease in integrin-β1 led to reduced ILK expression, and subsequently contributed to the upregulation and downregulation of the EMT-related molecules E-cadherin and Snail, respectively. Concomitantly, analysis of other integrin related molecules resulted in a decrease in phosphorylated focal adhesion kinase (pFAK), which is a cytoplasmic tyrosine kinase that plays critical roles in integrin-mediated signalling. Furthermore, results of immunoprecipitation assays using an integrin-β1 antibody indicated a decreased tendency of Talin and vinculin binding, which is considered important for establishing an activated form of integrin on the cell surface (Supplementary Fig. S3a and b). Collectively, these findings suggest that ERO1α regulates cancer progression by activating integrin signalling under hypoxia.

If this work is considered in the most critical terms, the possibility that false-positive results were obtained in the study owing to off-target effects cannot be entirely eliminated. However, a previous study reported that perfectly coinciding off-target sequences that are 13 or more bases upstream of the NGG sequence are likely to be cleaved^26^, but the off-target sequences in this case that were more than 13 bases upstream of the NGG sequence were not perfectly matched. Furthermore, the rescued ERO1α clone exhibited a recovery of tumourigenicity. In addition, as an alternative method, ERO1α knockdown (KD) clones using shRNA were prepared (Supplementary Fig. S4a). Similar to the results obtained with KO clones, the reduced cell surface localization and the change in the modification of integrin-β1 were confirmed by ERO1α KD (Supplementary Fig. S4b and c). Therefore, it is considered that the possibility of such off-target effects is extremely low.

Integrins are critical molecules in tumour growth and have thus attracted attention as therapeutic targets for cancer^27, 28^. Recently, studies using integrin-β1 KO MDA-MB-231 breast cancer cells^9^ and exogenously-introduced or RNAi-inhibited integrin-β1 in HT-29 cells^29^ have reported that there is a positive correlation between integrin-β1 expression and cancer cell proliferation. However, most integrin-KO mice exhibit embryonic or perinatal lethality^30^ and therefore, targeting integrins could produce adverse effects by inhibiting diverse interactions required for homeostasis. Conversely, ERO1α is ubiquitously and weakly expressed in normal cells, whereas KO mice develop normally because of compensation by other oxidizing enzymes (e.g., PRDX4, vitamin K epoxide oxidoreductase, and quiescin sulfhydryl oxidase)^31–36^.

In conclusion, our findings suggest that among oxidizing enzymes, ERO1α is predominantly activated only under abnormal conditions such as hypoxia and contributes to cancer progression, and further that ERO1α might serve as an optimal target for cancer therapy because ERO1α suppression could inhibit integrin-related signalling specifically in the cancer microenvironment. Here, we have revealed a previously unrecognized pathway through which ERO1α can potentially modulate tumour cell behaviour under hypoxia and thus favour cancer progression. Collectively, the findings of this study involving a gene-modification approach indicate that ERO1α is a major proliferation-enhancing factor in cancer cells and could be used as both a diagnostic biomarker of cancer exacerbation and a therapeutic target.

## Methods

### Cell culture

The human colorectal cancer HCT116 cell line was purchased from American Type Culture Collection. The cells were cultured in DMEM (Sigma-Aldrich) supplemented with 10% foetal bovine serum (FBS; Gibco) and 100 U/mL each of penicillin and streptomycin. All cells were incubated at 37 °C in a humidified atmosphere containing 95% air and 5% CO_2_.

### Establishment of ERO1α KO and ERO1α-rescued clones derived from the HCT116 colorectal cancer cell line

The pCG SapI vector, which is an all-in-one vector for CRISPR/Cas9 genome editing, contains the Cas9 sequence and a cloning site for targeted gRNA sequence insertion; the vector was a gift from Dr. Sakurai^37, 38^. The gRNA sequence designed specifically for ERO1α exon 1 that was inserted into the pCG vector was 5′-GCTCAGCTCGGGCCACGGAGAGG-3′. Subsequently, the constructed targeting vector and pcDNA3.1(+) were transfected into the HCT116 cell line using Lipofectamine 3000 (Thermo Fisher Scientific), after which the cells were cultured under G418 drug selection (800 μg/mL). Single-cell clones were selected and evaluated by using the T7 endonuclease assay to detect indels (insertions/deletions) in the target alleles. Next, PCR amplification was performed using purified genomic DNA as a template and primers designed for sites near the target gene locus, and then the obtained PCR products were subcloned into pGEM Teasy (Promega); the generated clones were picked and sequenced using a 3100 Genetic Analyzer (ABI) to confirm the presence of indels in the target sequence of each allele. Lastly, elimination of ERO1α expression at the protein level in the selected clones was confirmed through western blotting (below). The control cell clone (mock) was generated by transfecting the empty vector. To establish the rescued cell line, pCMV6-ERO1α (Origene) was transfected into the KO clone and cells were cultured in medium containing 0.8 μg/mL puromycin for selection of single colonies.

### Cell-proliferation and wound-healing (scratch) assays

Cells were cultured in 6-well plates for the duration of each experiment. In the proliferation assay, cells were stained with trypan blue and then counted under a microscope. To quantify cell-migration ability, cells were spread and cultured until confluence, and then scratched using a 200-μL pipet tip, washed with PBS, and incubated in fresh culture medium. After culturing for 24 h, the scratch space was analysed under a DP2-BSW microscope (Olympus).

For testing the effects of the hypoxic environment, cells were cultured in a CO_2_ incubator (1% O_2_/94% N_2_/5% CO_2_) for various durations, and at each time point, cells were either used in the proliferation assay or collected using a scraper for western blotting. From the collected cells, protein samples were obtained using TNE lysis buffer (1% Nonidet P-40, 50 mM Tris-HCl, pH 8.0, 150 mM NaCl, 1 mM EDTA) containing Protease Inhibitor Cocktail (Roche Diagnostics).

### Isolation and detection of biotinylated cell-surface proteins

To isolate biotinylated cell-surface proteins, cultured cells were washed with ice-cold PBS and then incubated with 0.5 mg/mL EZ-Link Sulfo-NHS-LC-biotin (Thermo Fisher Scientific) in PBS for 30 min on ice. The reaction was stopped by washing the cells with PBS containing 50 mM glycine. The surface-biotinylated cells were lysed in 400 μL of RIPA buffer (50 mM Tris, pH 8.0, 150 mM NaCl, 0.5% deoxycholate, 1% Nonidet P-40, 0.1% SDS, 1 × Protease Inhibitor Cocktail) and equal volumes of the biotinylated-protein solutions were bound to immobilized NeutrAvidin agarose (Thermo Fisher Scientific). The bound proteins were washed and then eluted by boiling in sample buffer for western blotting. The absence of α-tubulin in the NeutrAvidin-bound fraction was used to confirm that intracellular proteins were not biotinylated.

### Enzymatic deglycosylation

To confirm integrin glycosylation, cell lysates were denatured by heating at 100 °C for 10 min in glycoprotein denaturing buffer (New England Biolabs) and then incubated for 1 h at 37 °C with PNGase F or Endo H. Endo H cleaves N-linked glycoproteins and removes the chitobiose core of mannose and certain hybrid oligosaccharides. PNGase F is an amidase that removes all saccharide moieties. After the reaction, samples were analysed by western blotting with target-specific antibodies.

### Western blotting

The cells from each group were washed twice with PBS following culture for various times for each experiment and then scraped in a lysis buffer, TNE, or RIPA, containing phosphatase and protease inhibitors (Roche Diagnostics). The lysates were centrifuged and the collected supernatants were used for western blotting, which was performed according to procedures described previously^39^. Briefly, cell lysates were separated using SDS-PAGE (Wako) and transferred onto polyvinylidene difluoride membranes (Millipore) by using standard techniques (Bio-Rad). The immobilized proteins were blocked in sterilized 0.5% non-fat dry milk in PBS containing 0.05% Tween-20 (PBST) and then incubated overnight at 4 °C with primary polyclonal antibodies. The primary antibodies that were used are as follows: anti-ERO1α (Abnova), anti-integrin-β1, anti-HIF-1α (BD Biosciences), anti-ERO1β (Proteintech Group), anti-PDI, anti-ILK1, anti-Snail, anti-E-cadherin, anti-α-tubulin (Cell Signaling Technology), anti-PRDX4, anti-GAPDH, and anti-β-actin (Abcam). The membranes were washed and incubated with a secondary antibody (HRP-conjugated goat anti-mouse or -rabbit antibody; CST), as per manufacturer instructions, and then washed repeatedly with PBST before visualizing the immunoreactive complexes by using ECL reagent (Thermo Fisher Scientific) and a Chemidoc Imaging System (Bio-Rad). All experiments were repeated thrice. Uncropped scans are shown in Supplementary information.

### Xenograft model to examine *in vivo* tumourigenicity

BALB/c nu/nu mice (6–8 weeks old) were obtained from CLEA (Tokyo, Japan). All animal studies were approved and conducted in compliance with the guidelines of the Animal Studies Committee of Hokkaido University. For tumour-formation studies, mice were injected with 1 × 10^6^ WT HCT116 (control), mock control (mock), or ERO1α−/− clone (KO) cells into the lateral subcutaneous region. Tumour growth was measured once or twice a week in two dimensions by using callipers, and tumour volume (mm^3^) was calculated using this formula: volume = 3.14 × (width^2^ × length)/6.

Mice were euthanized when the tumour size reached 2000 mm^3^. At Day 28 after transplantation, tumours were excised, weighed, digitally imaged, and then stored in 10% formalin for histological analysis. Cell-proliferation curves were generated and then analysed using a Student’s *t* test. The difference between control and KO was statistically significant (*P* < 0.001).

### Immunohistochemistry

Formalin-fixed paraffin-embedded xenograft tumour samples were sectioned, deparaffinized, and incubated in pH 9 buffer (Nichirei Biosciences) at 105 °C for 15 min. After washing and blocking, each section was incubated with rabbit anti-human ERO1α (EPR12474, Abcam) primary antibody overnight at 4 °C and then with anti-rabbit IgG (H + L) Goat IgG Fab’-HRP (IBL, Gunma, Japan) for 45 min, and subsequently developed using DAB (3,3α-diaminobenzidine tetrachloride) for bright-field microscopy.

### Immunocytochemistry

Cells were cultured on 8-well chamber slides (BD Biosciences). After 24 h of culture, the cells were incubated under normoxia or hypoxia for 48 h, and then washed thrice with ice-cold PBS and fixed in 4% paraformaldehyde in PBS (Wako). After washing thrice more with ice-cold PBS, the cells were permeabilized with 0.1% Triton X-100 in PBS at room temperature for 5 min and incubated in 5% normal goat serum in PBS for blocking nonspecific staining. The sections were next incubated (4 °C, overnight) with mouse anti-human integrin-β1 (clone P5D2, Abcam) and rabbit anti-human PDI (C81H6, CST) primary antibodies, washed with PBS, and then reacted with AlexaFluor-488-conjugated goat anti-mouse IgG and AlexaFluor-555-conjugated goat anti-rabbit IgG secondary antibodies (Life Technologies). The specimens were mounted with ProLong Diamond Antifade Mountant with DAPI (Thermo Fisher Scientific). Lastly, sections were imaged using a fluorescence microscope, with the same imaging conditions used for all samples.

### Flow cytometry

Cells were cultured under a normoxic or hypoxic atmosphere for 48 h and then detached using a cell-dissociation buffer (Gibco) and fixed in 1% paraformaldehyde. The collected cells were passed through a cell strainer (BD Falcon) to generate single-cell suspensions in 200 μL of PBS containing 5% FBS and 0.05% NaN_3_ (FACS buffer). Next, the cells were stained with APC-labelled anti-human CD29 antibody for 30 min on ice, washed twice, resuspended in FACS buffer, and examined on a FACS Versa (BD Biosciences). Fluorescence compensation was performed using APC-conjugated mouse IgG1κ isotype control.

### Statistical analysis

The mean response of each experimental group was compared with its concurrent control by performing a Student’s *t* test. *P* < 0.05 was considered statistically significant.

## Electronic supplementary material

Supplementary information
