# Supplementary material for: Hypoxia-inducible ERO1α promotes cancer progression through modulation of integrin-β1 modification and signalling in HCT116 colorectal cancer cells
Source: Sci Rep. 2017 Aug 24;7:9389. doi: 10.1038/s41598-017-09976-7 (PMC5571208; doi:10.1038/s41598-017-09976-7)

Supplementary Information

Hypoxia-inducible ERO1α promotes cancer progression through modulation of integrin-β1 modification and signalling in HCT116 colorectal cancer cells

Norio Takei[1, *], Akihiro Yoneda[1], Kaori Sakai-Sawada[1], Marina Kosaka[1, 2], Kenjiro Minomi[2] & Yasuaki Tamura[1]

[1]Department of Molecular Therapeutics, Center for Food and Medical Innovation, Institute for the Promotion of Business-Regional Collaboration, Hokkaido University, Kita-21 Nishi-11, Kita-ku, Sapporo 001-0021, Japan.

[2]Research & Development Department, Nucleic Acid Medicine Business Division, Nitto Denko Corporation.

[*]Correspondence and requests for materials should be addressed to N.T. (e-mail: ntakei@fmi.hokudai.ac.jp)

Tel: 81-11-706-9464 (NT); Fax: 81-11-706-9463 (NT)

Supplementary Figure Legends

Supplementary figure S1. Gene expression analysis of *ITGB1* by microarray (a) and quantitative real-time (qRT)–PCR (b). The experimental procedure for cDNA microarray analysis was performed according to the manufacturer's instructions (Agilent Technologies). The relative amounts of mRNA were calculated from the comparative threshold cycle (Ct) values relative to *GAPDH*. There was no difference in integrin-β1 expression between WT and KO under conditions of either normoxia or hypoxia.

Supplementary figure S2. Confirmation of reproducibility using another colon cancer cell line, HT-29. (**a**) Western blot (WB) analysis of the generated ERO1α KO clone in the HT29 cell line in comparison to the WT control. We used the experimental clone.8 and clone.10 as KO-1, KO-2, respectively. (**b**) Xenograft tumour growth analysis. Tumour size was measured twice a week after subcutaneous injection. KO clones showed significant inhibition of tumourigenesis ($n = 4$, *$P < 0.05$ by two-tail unpaired t-test. (**c**) Tumour weights were measured after dissection. Tumour values are given as the means ± S.E ($n = 4$) *$P < 0.05$ by Student's t-test. n.s. is not significant. (**d**) Tumour images in the indicated clone after 28 days. (**e**) Expression levels of membrane integrin-β1 on HCT116 clones under normoxia or hypoxia were confirmed by flow cytometric analysis. Compared with WT, attenuation of cell surface integrin-β1 was observed in KO. ERO1α KO in the HT-29 cell line showed the same phenotype as in HCT 116 cells.

Supplementary figure S3. ERO1α deletion affects integrin signal molecules under hypoxia. (**a**) WB analysis of other integrin signal molecules in WT, mock, and KO clones in HCT116 under each culture condition. In addition to the decrease in ILK-1 mentioned in Fig. 5b, a decrease in pFAK was also observed in KO clones. (**b**) Immunoprecipitation (IP) using hypoxia cell lysates with integrin-β1 antibody. Input and immunoprecipitates were subjected to WB analysis using indicated antibodies. In KO clones, decrease of Talin and vinculin binding amount was observed.

Supplementary figure S4. Determination of off-target effects. To exclude the possibility of off target effects, we established ERO1α KD cell clones in HCT116 with two ERO1α shRNAs, another gene silencing method. (**a**) WB analysis of generated ERO1α shRNA clones in HCT116 cell line in comparison with shcontrol shERO1α clones. We used clone 5 of each of the shRNAs for the experiment. (b) The expression level of membrane integrin-β1 on HCT116 clones under normoxia or hypoxia was confirmed by flow cytometric analysis. (c) WB analysis using cell lysates of each clone under normoxia and hypoxia. In KD clones using ERO1α shRNA No. 2 with ERO1α 2 in which ERO1α KD was established even under hypoxic conditions, the cell surface localization of integrin-β1 was observed to be decreased under hypoxia and the change in Integrin-β1 modification was clearly confirmed by WB. This is consistent with the result of KO clones using CRISPR/Cas9. FACS analysis was conducted twice in triplicate. Representative images are shown.

Supplementary figure S1

a

			raw data				standardization				calculation of the difference between the expression level	Gene expression levels		
ProbeName	GeneName	SystematicNa	WT N	WT Hy	KO N	KO Hy	WT N	WT Hy	KO N	KO Hy	Gene expression (KO-WT hypoxia)	KOhy-WThy	Log fold change	Fold change
A_23_P144369	ITGB1	NM_133376	843.4485	958.3588	579.8495	1225.864	0.873846	1.061743	0.886203	1.160379	0.09863651	0.09863651	0.394657936	1.314631024
A_24_P348845	ITGB1	NM_133376	886.2055	958.7776	634.6627	1328.768	0.891604	1.061902	0.918904	1.189458	0.127556058	0.127556058	0.510368937	1.424414412
A_23_P110052	ITGB1	NM_133376	919.4572	978.9245	599	1211.546	0.904832	1.069467	0.897967	1.156141	0.086673855	0.086673855	0.346793745	1.27173118
A_23_P133868	ITGB1	NM_133376	926.5698	981.7379	610.9073	1225.159	0.9076	1.070511	0.905093	1.160172	0.08966057	0.08966057	0.358743995	1.282309038
A_33_P3305885	ITGB1	NM_133376	937.6373	933.896	630.7993	1223.83	0.911864	1.052336	0.916694	1.15978	0.107444342	0.107444342	0.429899258	1.347139504
A_24_P303145	ITGB1	NM_133376	870.1378	913.7929	543.6239	1274.188	0.885034	1.044419	0.862848	1.174327	0.129907799	0.129907799	0.519778566	1.433735173
A_24_P618401	ITGB1	NM_133376	891.3415	929.1382	566.1449	1298.636	0.89368	1.050478	0.877544	1.181183	0.130705338	0.130705338	0.522969628	1.436909926
A_23_P106727	ITGB1	NM_133376	850.3101	886.5001	570.4923	1243.769	0.876756	1.033388	0.880313	1.16561	0.132222605	0.132222605	0.529040419	1.442969113
A_33_P3298128	ITGB1	NM_133376	843.0092	874.5552	557.7748	1181.018	0.873659	1.028452	0.872152	1.146935	0.11848218	0.11848218	0.474063132	1.389015918
A_24_P945000	ITGB1	NM_133376	920.226	947.7835	692.7351	1218.62	0.905133	1.057706	0.950602	1.158241	0.100535224	0.100535224	0.402254946	1.321571927
A_33_P3305885	ITGB1	NM_133376	937.6373	933.896	630.7993	1223.83	0.911864	1.052336	0.916694	1.15978	0.107444342	0.107444342	0.429899258	1.347139504

N :normoxia
Hy:hypoxia

b

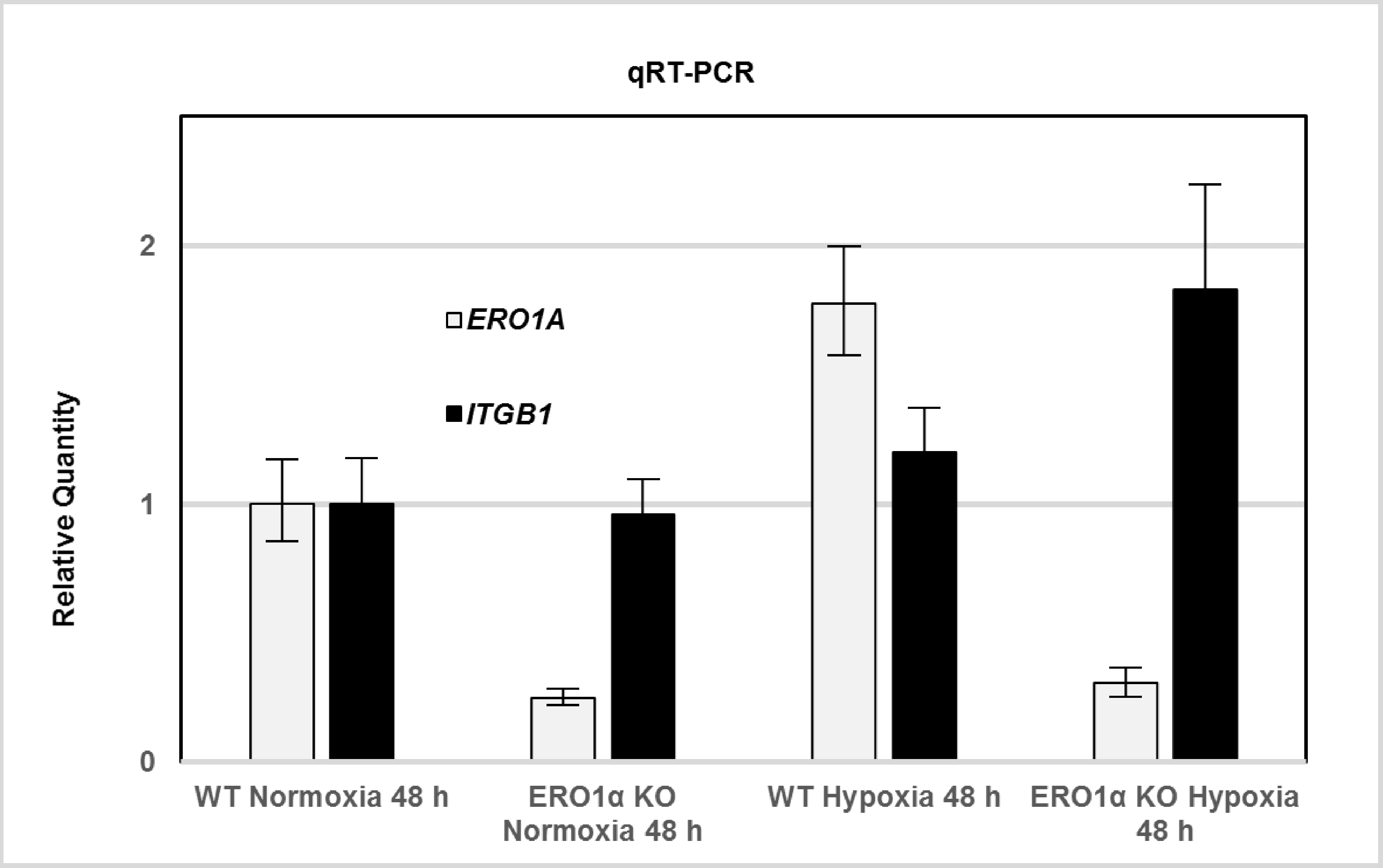

Supplementary figure S2

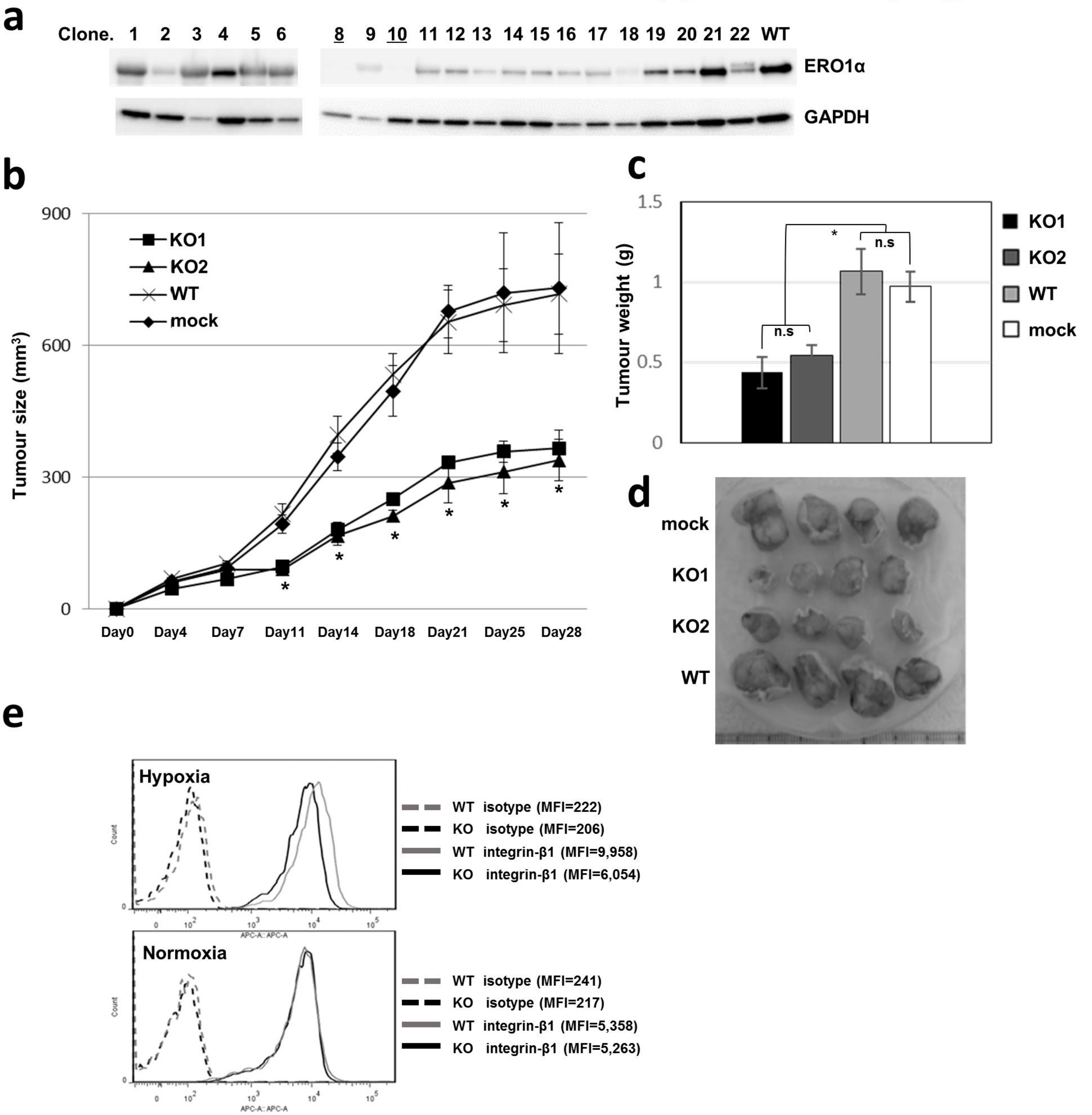

Supplementary figure S3

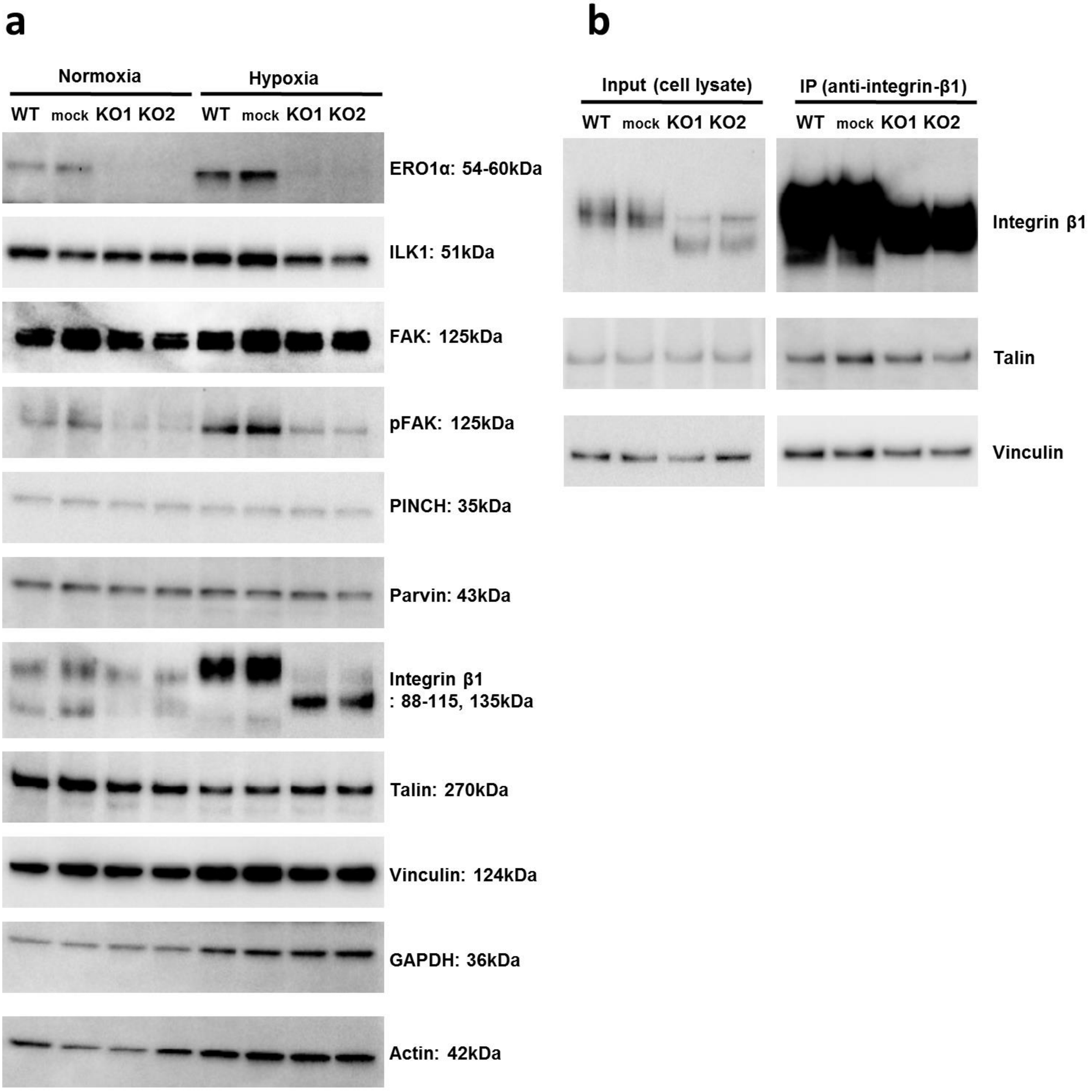

Supplementary figure S4

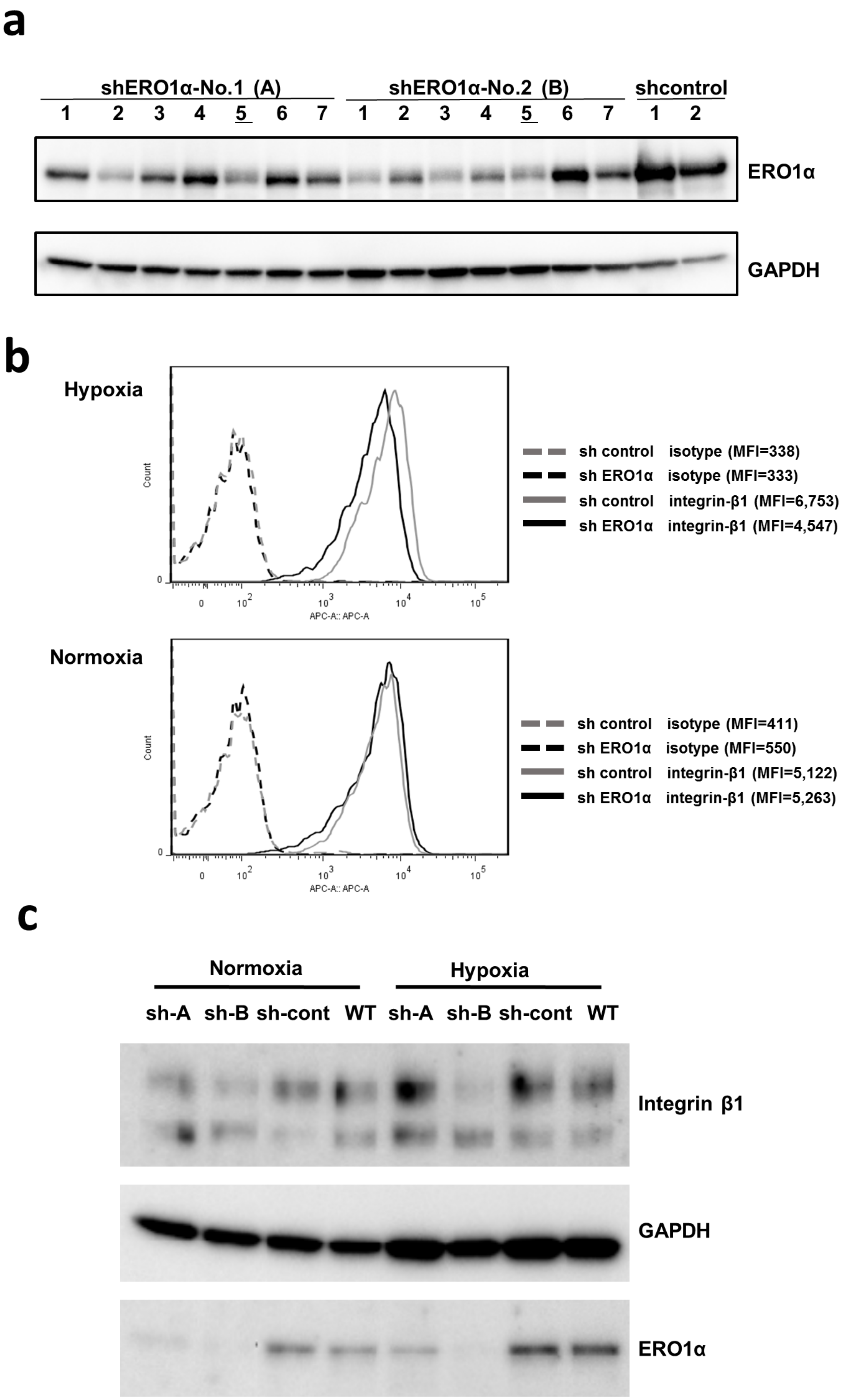

Supplementary information: Uncropped blots used in figures

Figure 1d

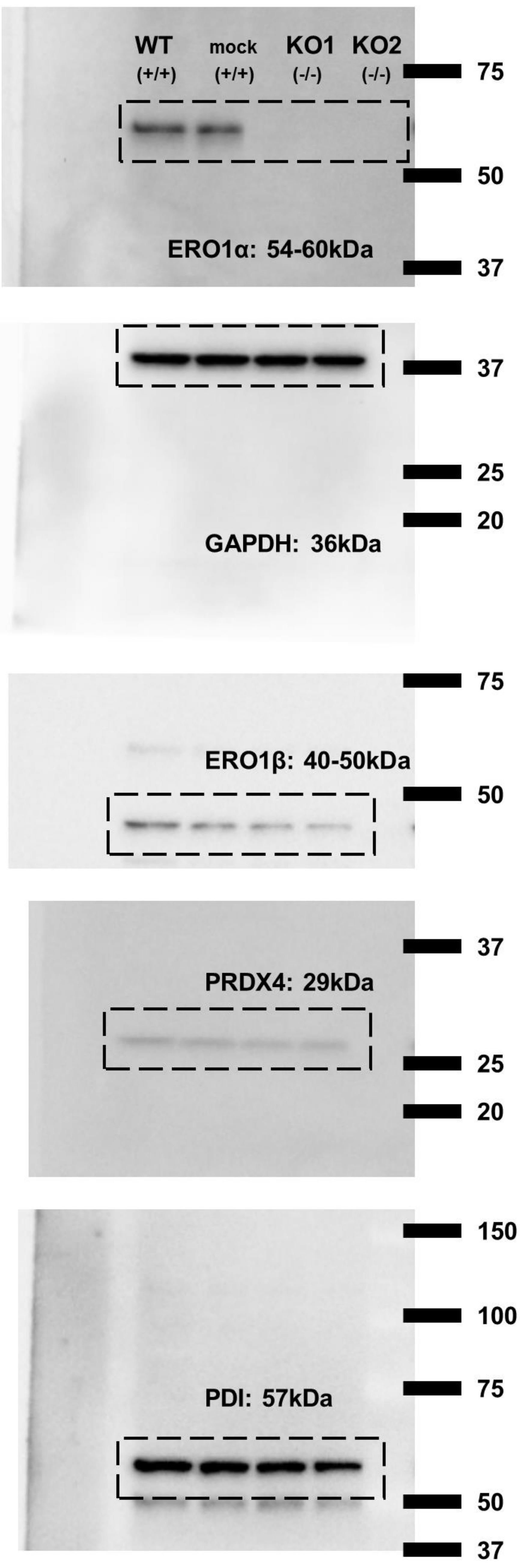

Figure 3c

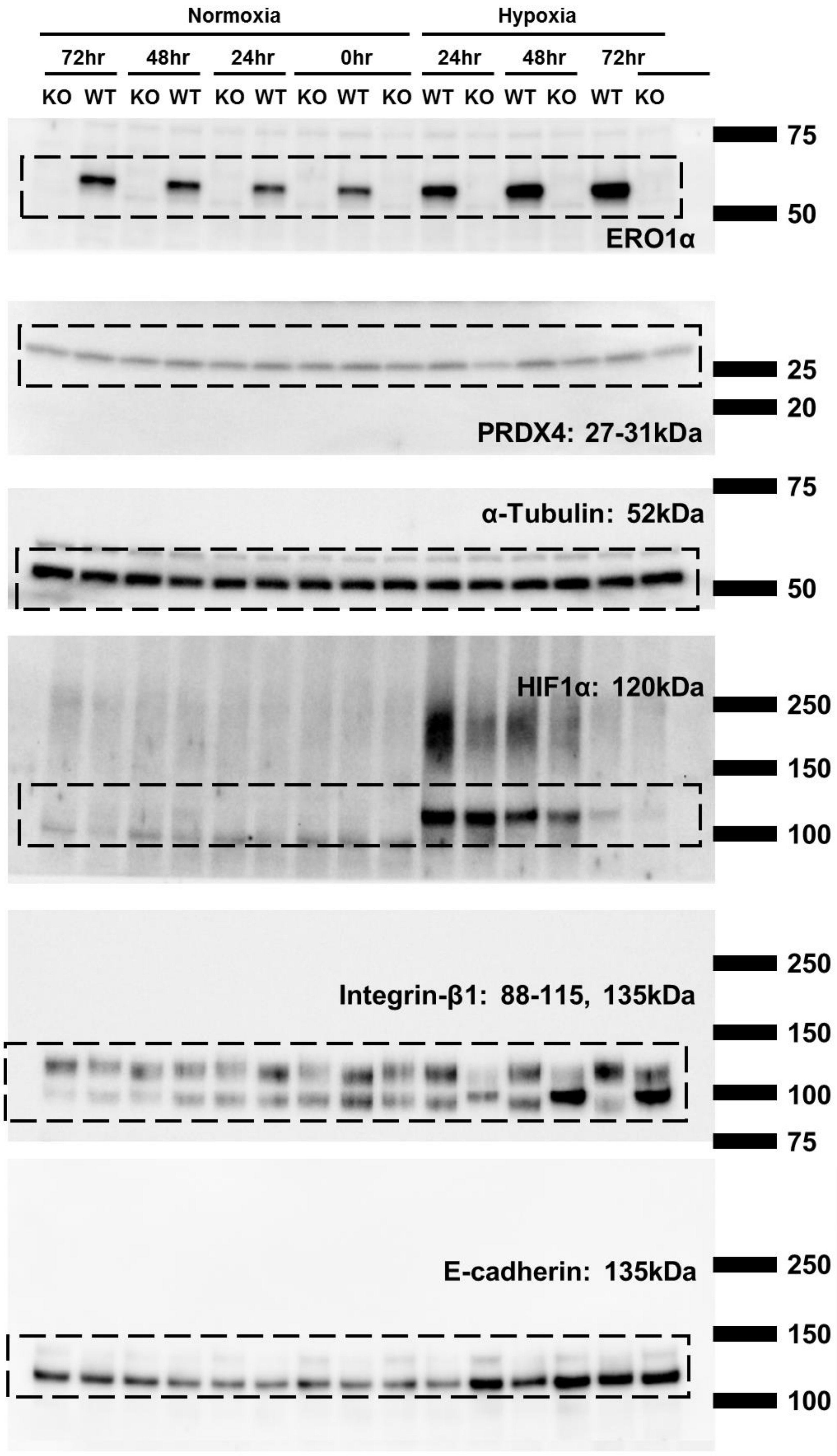

Figure 4a

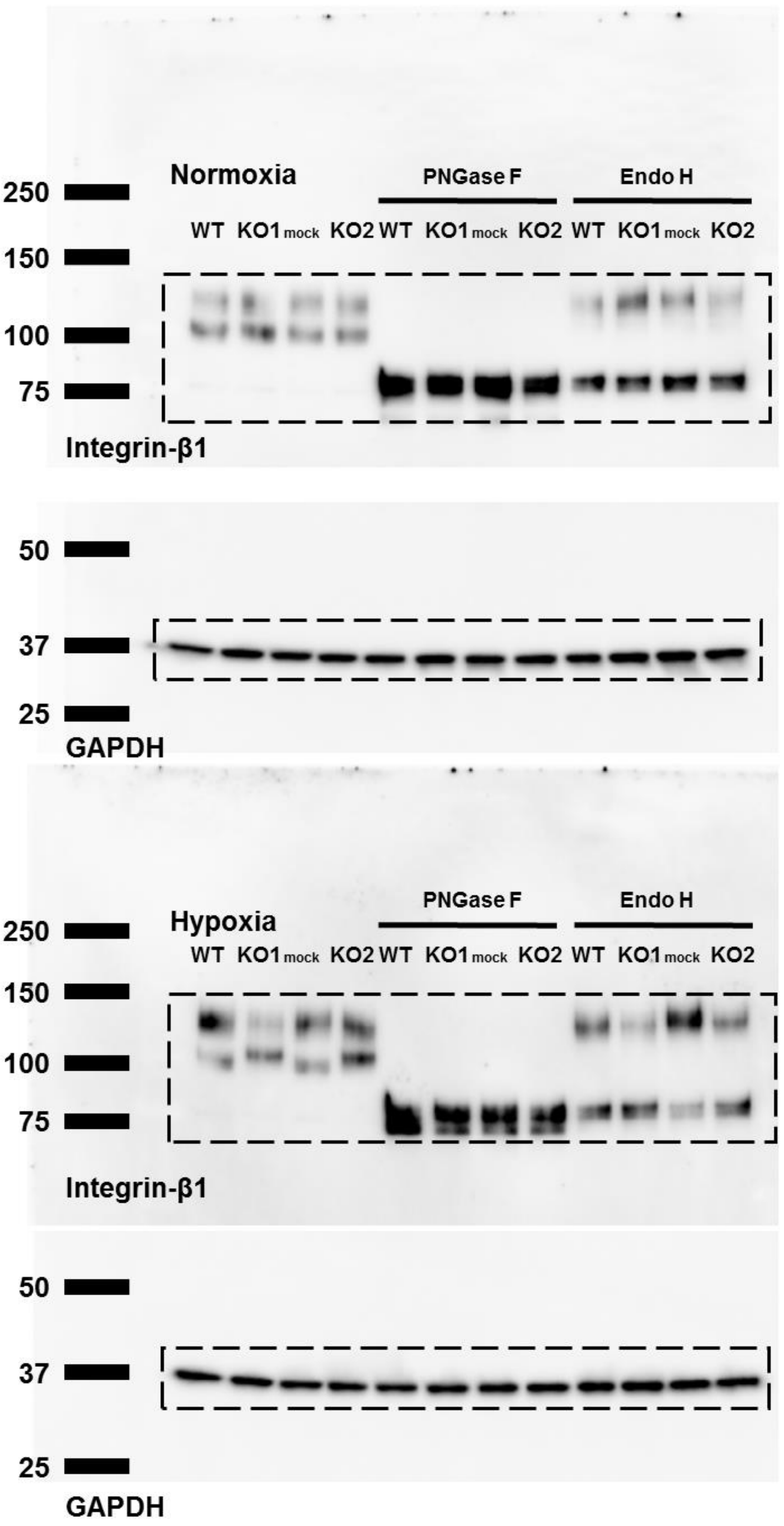

Figure 4b

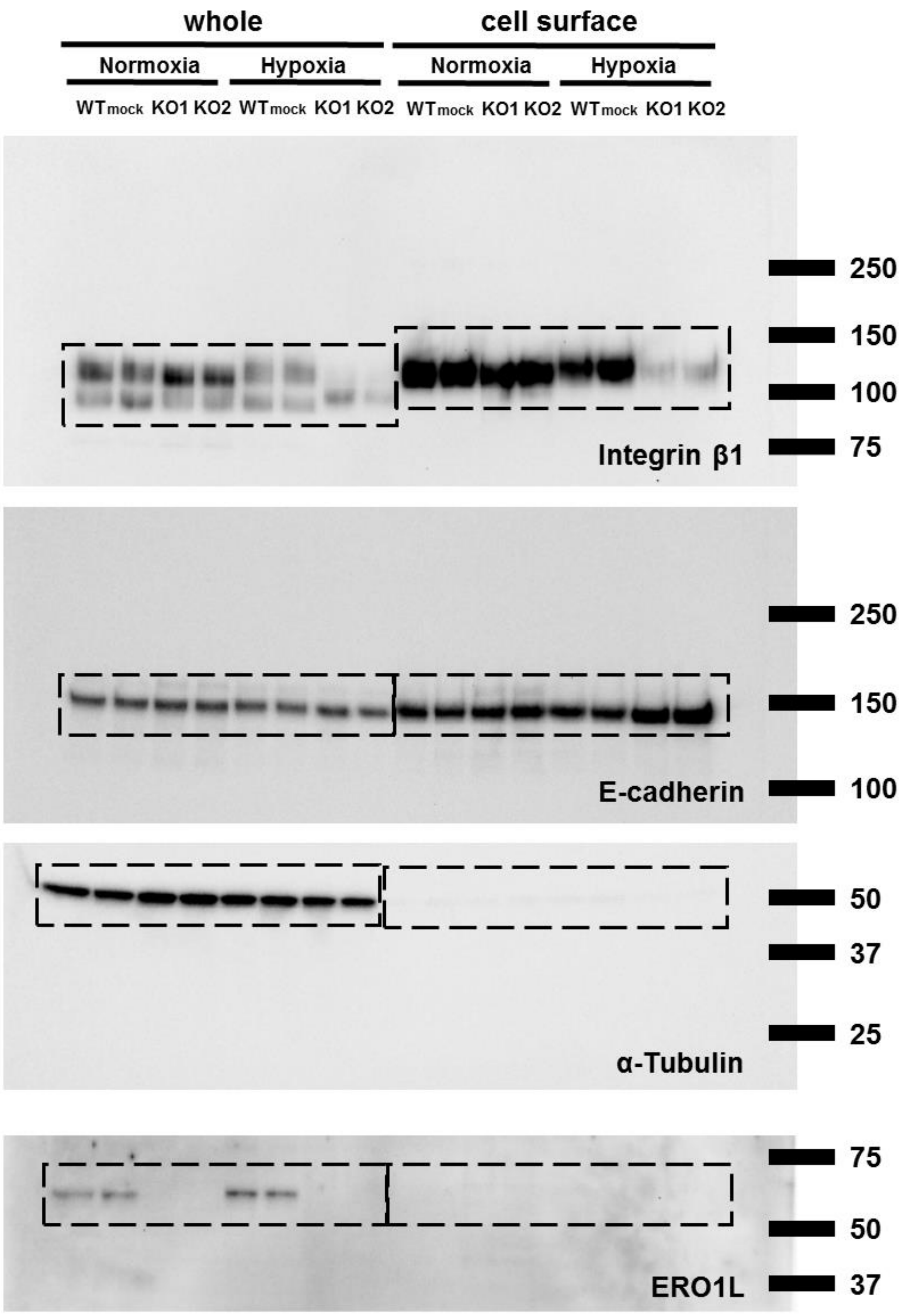

Figure 5b

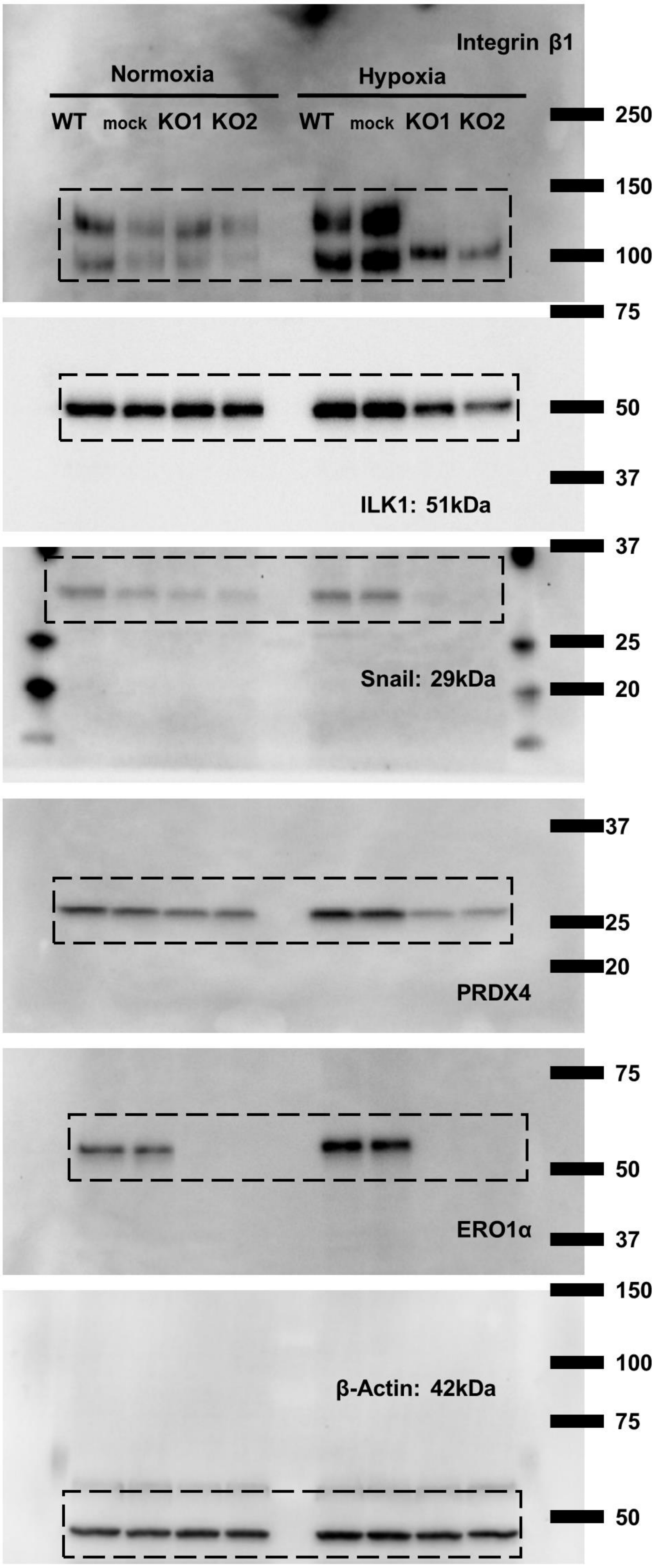

Figure 6b

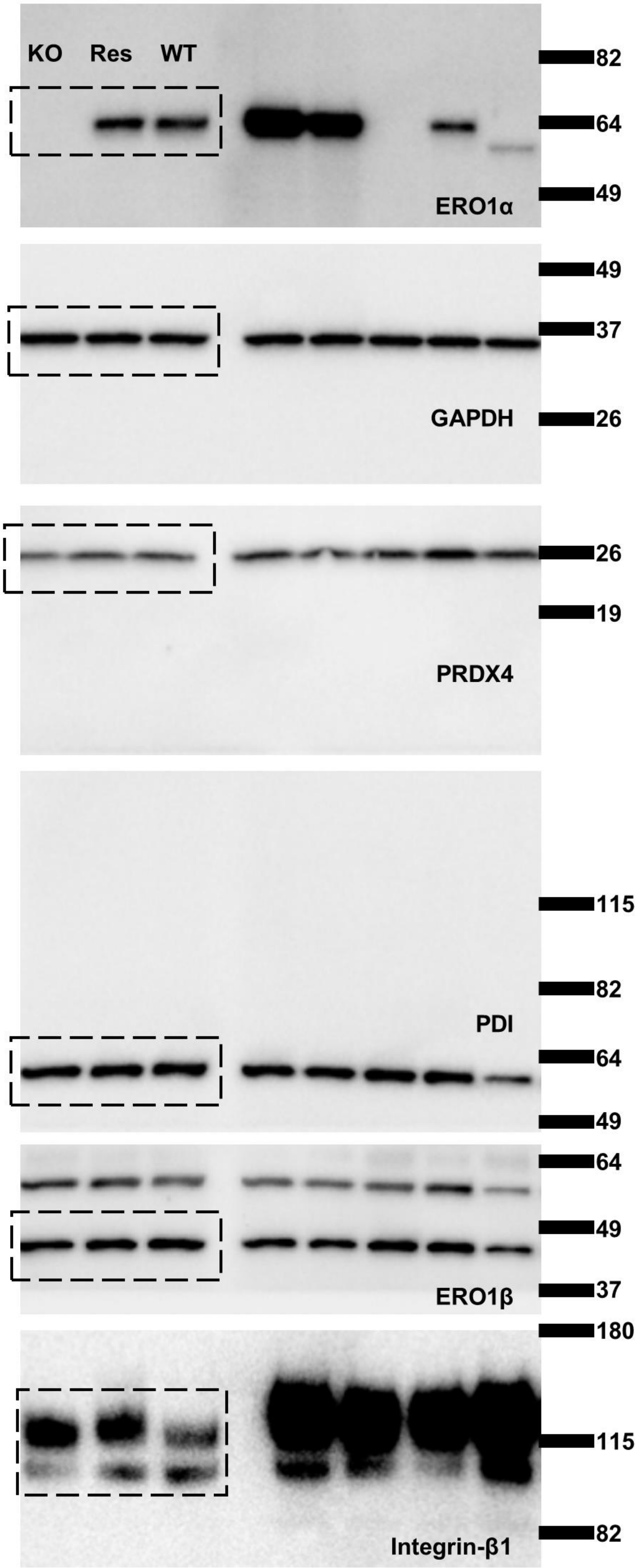

Supplement: Supplementary file 1 — Supplementary information [file 41598_2017_9976_MOESM1_ESM.pdf]
